# Identification of three tumor antigens and immune subtypes for mRNA vaccine development in diffuse glioma

**DOI:** 10.7150/thno.61677

**Published:** 2021-10-03

**Authors:** Quanwei Zhou, Xuejun Yan, Hecheng Zhu, Zhaoqi Xin, Jin Zhao, Wenyue Shen, Wen Yin, Youwei Guo, Hongjuan Xu, Ming Zhao, Weidong Liu, Xingjun Jiang, Caiping Ren

**Affiliations:** 1 Department of Neurosurgery, Xiangya Hospital, Central South University, Changsha, Hunan Province, 410008, China.; 2 Cancer Research Institute, School of Basic Medical Science, Central South University, Changsha, China.; 3 Changsha Kexin Cancer Hospital, Changsha, Hunan 410205, China.; 4 The NHC Key Laboratory of Carcinogenesis and the Key Laboratory of Carcinogenesis and Cancer Invasion of the Chinese Ministry of Education, Xiangya Hospital, Central South University, Changsha, China.

**Keywords:** diffuse glioma, tumor antigen, immune subtypes, mRNA vaccine, tumor immune infiltration

## Abstract

**Rationale:** Diffuse glioma patients have high mortality and recurrence despite multimodal therapies. This study aims to identify the potential tumor antigens for mRNA vaccines and subtypes suitable for the immunotherapy of patients with diffuse glioma.

**Methods:** Gene expression profiles and corresponding clinical information were obtained from the Chinese Glioma Genome Atlas (CGGA) and the Cancer Genome Atlas (TCGA) databases. Genetic alterations were extracted from cBioPortal. Differential gene analysis, survival analysis, correlation analysis, consensus clustering analysis, and immune cell infiltration analysis were conducted based on the various databases. Finally, the hub genes, the modules related to tumor antigens, and the immune subtypes were identified using WGCNA method.

**Results:** Three over-expressed, amplified, and mutated tumor antigens, including KDR, COL1A2, and SAMD9, were associated with clinical outcomes. The expression of the three genes had a positive correlation with the abundance of antigen-presenting cells (APCs) and APC marker expression. Subsequently, three immune subtypes (Ims1, Ims2, and Ims3) were distinguished in the TCGA cohort, which exhibited distinct molecular, cellular, and clinical characteristics consistent with the CGGA cohort. Diffuse gliomas with subtype Ims1 were more malignant with immunosuppressive phenotypes and more associated with poor prognosis than the other two subtypes. The three antigens and the immune checkpoints were differentially expressed among the three immune subtypes. Finally, functional enrichment analysis of the genes related to tumor antigens and immune subtypes suggested that they are enriched in many immune-associated processes.

**Conclusions:** KDR, COL1A2, and SAMD9 are potential antigens for developing mRNA vaccines against diffuse glioma. The results suggest that immunotherapy targeting these three antigens is more suitable for patients with subtype Ims1. This study provides insights into immunotherapy for diffuse glioma.

## Introduction

With a median survival time of 1 to 15 years, diffuse glioma is the most aggressive and fatal type of primary brain tumors 1, 2. According to the distinct biological feature, diffuse gliomas are histologically classified into World Health Organization (WHO) grade II, grade III, and grade IV. Diffuse glioma also shows molecular heterogeneity in the mutational status of isocitrate dehydrogenase 1 and 2 (IDH1/2), the codeletion status of chromosome arms 1p and 19q (1p19q), and the promoter methylation of O(6)-methylguanine-DNA methyltransferase (MGMT) 3. In recent years, multimodal therapies, including maximum safe surgical resection assisted by radiation therapy and concurrent temozolomide (TMZ) chemotherapy, have been adopted to treat diffuse gliomas 4. However, the recurrence of diffuse gliomas, especially glioblastoma (GBM), is almost inevitable, and the prognosis of patients remains poor. The limited therapeutic effect and fast tumor progression might be attributed to the specific molecular characteristics and heterogeneous cellular components of diffuse gliomas. To date, therapies based on biological characteristics have failed to improve patient outcomes, and novel treatment strategies are needed for patients with diffuse gliomas.

Recently, immunotherapy has achieved considerable success 5. Immune checkpoint inhibitors targeting PD-1/PD-L1 and CTLA-4 have been developed and successfully adopted to treat melanoma and non-small cell lung cancer 6, 7. However, patients with diffuse glioma could not sufficiently benefit from cancer treatment using checkpoint inhibitors 8. mRNA vaccines have attracted the attention of scientists and oncologists and have become a hotspot in immunotherapy 9, 10. Tumor antigens produced by genetic or epigenetic aberrations in oncogenesis could provoke adaptive immune responses against tumor cells via surface molecular features 11. As the prognostic indicators and therapeutic targets of malignant tumors, tumor antigens can be subdivided into nonmutant proteins with tumor-associated expression, neoantigens (mutant proteins), and virus-derived antigens originating from the integration of infective viral genes into the cellular genome 12. Currently, next-generation sequencing, computational analysis, and immunopeptidomics help identify novel tumor antigens 13. In addition to surgery, radiotherapy, and chemotherapy, antigen-targeted immunotherapy has become a hotspot in the field of tumor treatment 10. Recently, mRNA vaccine therapeutics have been shown to be effective by several preclinical and clinical studies for melanoma 14, prostate cancer 15, and breast cancer 16. Glioma treatment targeting tumor-specific neoantigens has attracted significant attention. Personalized neoantigen-targeting vaccines based on mutations and transcriptome analyses were used to immunize GBM patients 9, 17. IDH1 frequently mutates in glioma, and a vaccine targeting the mutant IDH1 induces antitumor immunity resulted from mutation-specific anti-IDH1 (R132H) 18. In a clinical trial, patients treated with the H3.3K27M-specific vaccine showed prolonged overall survival (OS) 19.

This study aims to identify the potential tumor antigens for mRNA vaccine development and the immune subtypes to select suitable patients for diffuse glioma vaccination. Three over-expressed, amplified, and mutated molecules were identified as tumor antigens, i.e., KDR, COL1A2, and SAMD9, and found to be associated with unfavorable prognosis. These tumor antigens were markedly associated with APC infiltration in diffuse glioma. Three robust immune subtypes were then identified in the TCGA and CGGA cohorts based on the profiles of immune-related genes. Diffuse glioma with the three immune subtypes exhibited distinct cellular, molecular, and clinical characteristics in both cohorts. Notably, the three tumor antigens were highly expressed and found to be associated with APC marker expression in subtype Ims1 diffuse gliomas. The results revealed three antigens that could be used to develop mRNA vaccines, and the subtype Ims1 was identified as suitable for vaccination against diffuse glioma.

## Methods

### Data source and data processing

The normalized gene expression (n = 703), somatic gene mutations (MAF files, n = 1090), and clinical records (n=1105) of patients with diffuse gliomas were retrieved from the Cancer Genome Atlas (TCGA, https://tcga-data.nci.nih.gov/tcga/). In the TCGA cohort, a total of 677 samples, including nontumor samples (n = 5), grade II samples (n = 249), grade III samples (n = 262), and grade IV samples (n = 161), were selected from 703 cases by averaging the values from the same patient and excluding samples. Additionally, the validated cohort was downloaded from the Chinese Glioma Genome Atlas (CGGA) dataset (n = 693, http://www.cgga.org.cn) 20.

Gene copy number variation (CNV) data were collected from cBioPortal for Cancer Genomics (cBioPortal, http://www.cbioportal.org) to identify the amplified genes. In addition, tumor mutational burden (TMB) and mutation counts were obtained from the somatic mutation frequencies. The “outliers” package was used to exclude extreme deviate values.

### Gene differential expression analysis

A total of 3982 over-expressed genes in lower-grade glioma and 5224 over-expressed genes in GBMs with log_2_ (fold change) values more than 1 and *P* values less than 0.01 were obtained and the LIMMA method was used for the Gene Expression Profiling Interactive Analysis (GEPIA, http://gepia2.cancer-pku.cn) 21, which combined the TCGA dataset and the Genotype-Tissue Expression (GTEx) database.

### Identification of subtypes by immune-related genes in diffuse glioma

A total of 3126 immune-related genes extracted from the Gene Ontology (GO) database were used for Consensus Cluster Plus analysis 22. The optimal cluster number was determined by the cumulative distribution function curves (CDF) of the consensus score. A t-distributed stochastic neighbor embedding (*t*-SNE)-based approach was then used to validate the subtype assignments using the mRNA expression data.

### Survival analysis

Samples with no more than 30 days of OS, disease-specific survival (DSS), or progression-free interval (PFI) were excluded from the TCGA and CGGA cohorts. The Kaplan-Meier method was used to assess OS, PFI, or DSS of the patients with immune subtypes and low- and high-expression groups of over-expressed genes in the TCGA and CGGA cohorts. The log-rank test was used to assess statistical significance using the R package “survival” 23. A *P*-value < 0.05 was considered statistically significant.

### Estimation of immune and stromal infiltration

The abundance of APCs was obtained from Tumor Immune Estimation Resource (TIMER, https://cistrome.shinyapps.io/timer/) 24. In addition, single-sample Gene Set Enrichment Analysis (ssGSEA) was used to calculate the relative abundance of 35 immune cell types for each sample 25. The gene list of each immune cell type was obtained from recent publications 26-28.

### Functional enrichment analysis

Genes with Pearson correlation coefficients over 0.3 (*P* < 0.05) were identified as those associated with the three antigens. Weighted gene coexpression network analysis (WGCNA) was conducted to identify the hub genes and modules associated with the three tumor antigens and immune subtypes 29. The functional enrichment analysis of hub genes was annotated by the Database for Annotation, Visualization, and Integrated Discovery (DAVID). The GO categories included cellular components (CC), biological processes (BP), and molecular function (MF). *P* < 0.05 was defined as the cutoff criterion.

### Statistical analysis

Unpaired Student's* t*-test was used to compare the two groups with distributed variables. One-way analysis and Kruskal-Wallis tests of variance were adopted as parametric and nonparametric methods, respectively, for comparing multiple groups. Contingent variables were analyzed with the chi-square test or Fisher's exact test. Pearson's test or Spearman's test was conducted to analyze the correlation between gene expression and the abundance of immune cells or gene expression. All statistical analyses were performed on GraphPad Prism 7.0 or R software (Version 3.6.0, https://www.r-project.org/). A two-tailed *P* value < 0.05 was considered statistically significant.

## Results

### Identification of potential antigens for diffuse glioma

**Figure 1:**
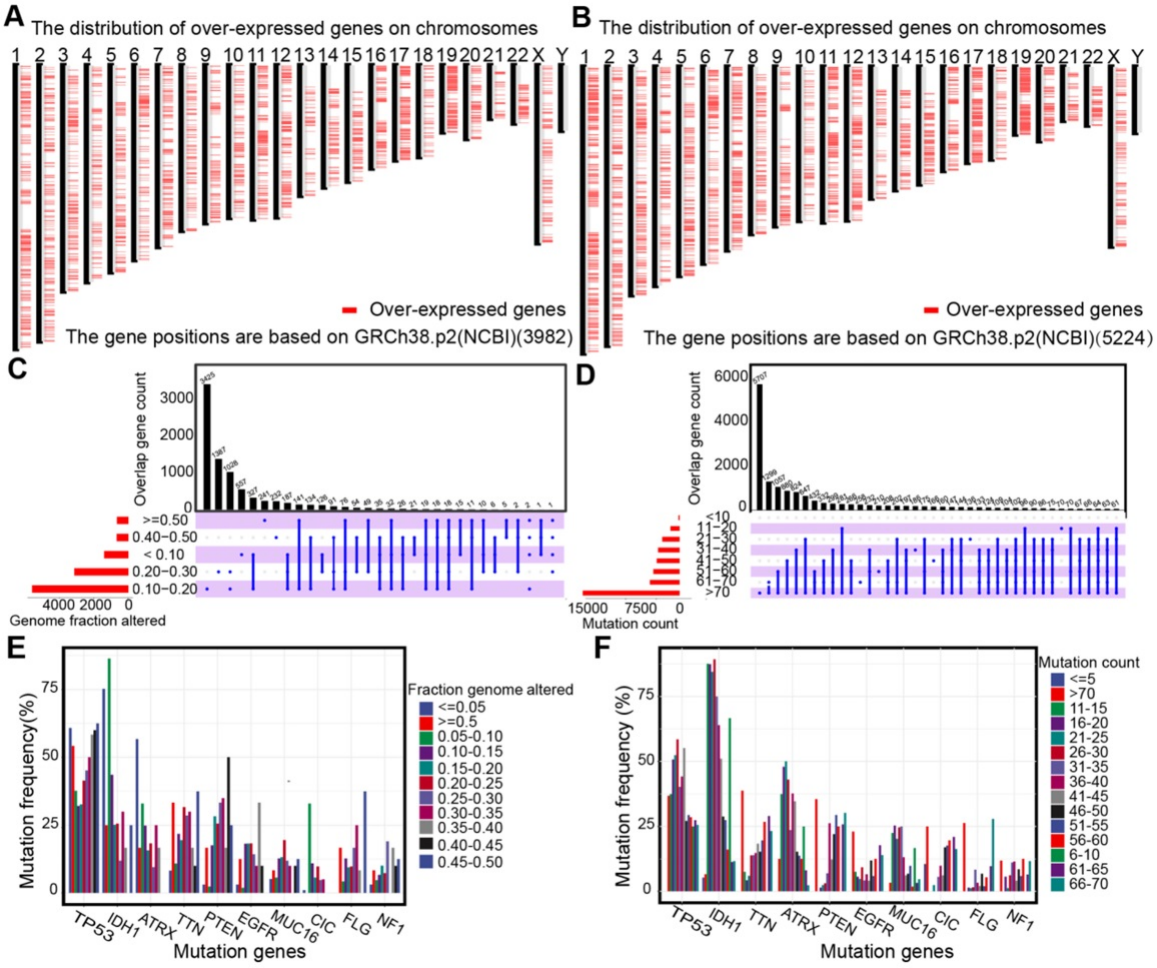
** Identification of potential tumor antigens in diffuse glioma. (A-B)** Chromosomal distribution of up-regulated genes in LGG (A) and GBM (B). **(C-F)** Identification of potential tumor-specific antigens of diffuse glioma. Genes overlapping in altered genome fraction (C) and mutation count groups (D). Genes with the highest frequency in the altered genome fraction (E) and mutation count groups (F).

The up-regulated genes that may encode tumor antigens were first selected among normal tissues, lower-grade gliomas, and higher grade gliomas to detect the potential antigens for diffuse glioma (Figure 1A-B). Then, the mutated genes were assessed by the altered genome fraction and mutation counts. Next, the frequently mutated genes that may encode tumor-specific antigens were selected by analyzing the altered genome fraction and mutation counts in each sample. The results showed that low fraction genome alterations and mutation counts were enriched in most patients with diffuse glioma, suggesting that most patients had low immunogenicity (Figure 1C-D). The ten most frequently mutated genes were identified, i.e., TP53, IDH1, TTN, ATRX, PTEN, MUC16, CIC, EGFR, FLG, and NF1, based on the altered genome fraction and mutation counts. Most of the ten genes with high alteration frequency were enriched in the highly altered genome fraction and mutation counts (Figure 1E-F). Finally, 2156 amplified genes were explored based on CNAs from the over-expressed genes. In conclusion, some potential antigens from those over-expressed, amplified, and mutated genes were identified in the TCGA dataset.

### Identification of clinical outcome-associated tumor antigens of diffuse glioma

**Figure 2:**
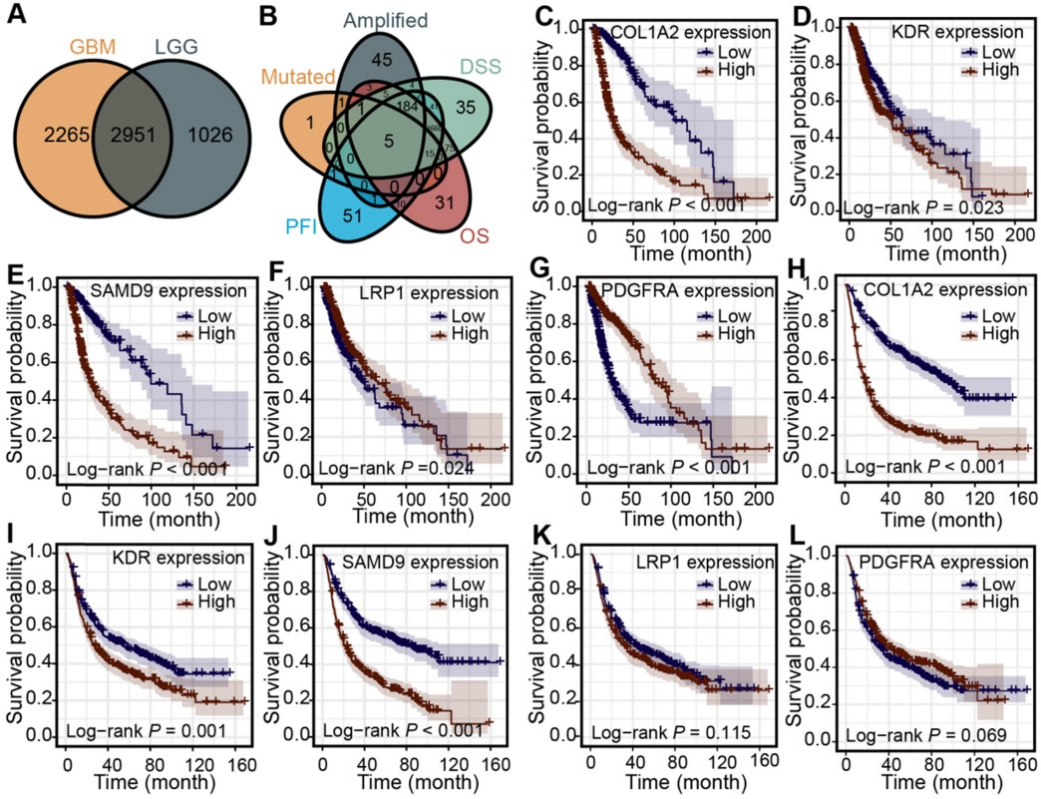
** Identification of tumor antigens associated with clinical outcome in diffuse glioma. (A)** Up-regulated genes overlapping in LGG and GBM. **(B)** Amplified and mutated tumor antigens significantly associated with OS, DSS, and PFI from up-regulated genes.** (C-G)** Survival analysis for COL1A2 (C), KDR (D), SAMD9 (E), LRP1 (F), and PDGFRA (G) in the TCGA cohort. **(H-L)** Survival analysis for COL1A2 (H), KDR (I), SAMD9 (J), LRP1 (K), and PDGFRA (L) in the CGGA cohort. The log-rank test was used to determine the statistical significance of the differences, and *P* < 0.05 was considered significant.

First, 2951 genes were derived from the overlap of differentially expressed genes from lower-grade gliomas and higher-grade gliomas (Figure 2A). The association between the mRNA expression of the over-expressed genes and patients' OS, PFI, and DSS was analyzed in the TCGA cohort to identify more clinically valuable tumor antigens of diffuse glioma. Five shared genes significantly associated with patient outcomes were selected, including KDR, PDGFRA, LRP1, COL1A2, and SAMD9, from the over-expressed, amplified, and mutated genes in the TCGA cohort (Figure 2B). The patients with high KDR, PDGFRA, LRP1, COL1A2, and SAMD9 expression had shorter OS (Figure 2C-G) (log-rank test, *P* < 0.05), indicating that tumor antigens were critical for the progression of diffuse gliomas. Diffuse gliomas with high expression of COL1A2, SAMD9, and KDR had poor prognoses in the CGGA cohort (Figure 2H-J) (log-rank test, *P* < 0.05), which was consistent with the TCGA cohort. However, statistically significant relationships were not observed between the mRNA expression of PDGFRA and LRP1 and the OS of patients in the CGGA cohort (Figure 2K-L) (log-rank test, *P* > 0.05). Therefore, three antigens associated with poor prognosis were identified for diffuse glioma in the TCGA and CGGA cohorts.

### The correlation between identified tumor antigens and the abundance of APCs in diffuse glioma

**Figure 3:**
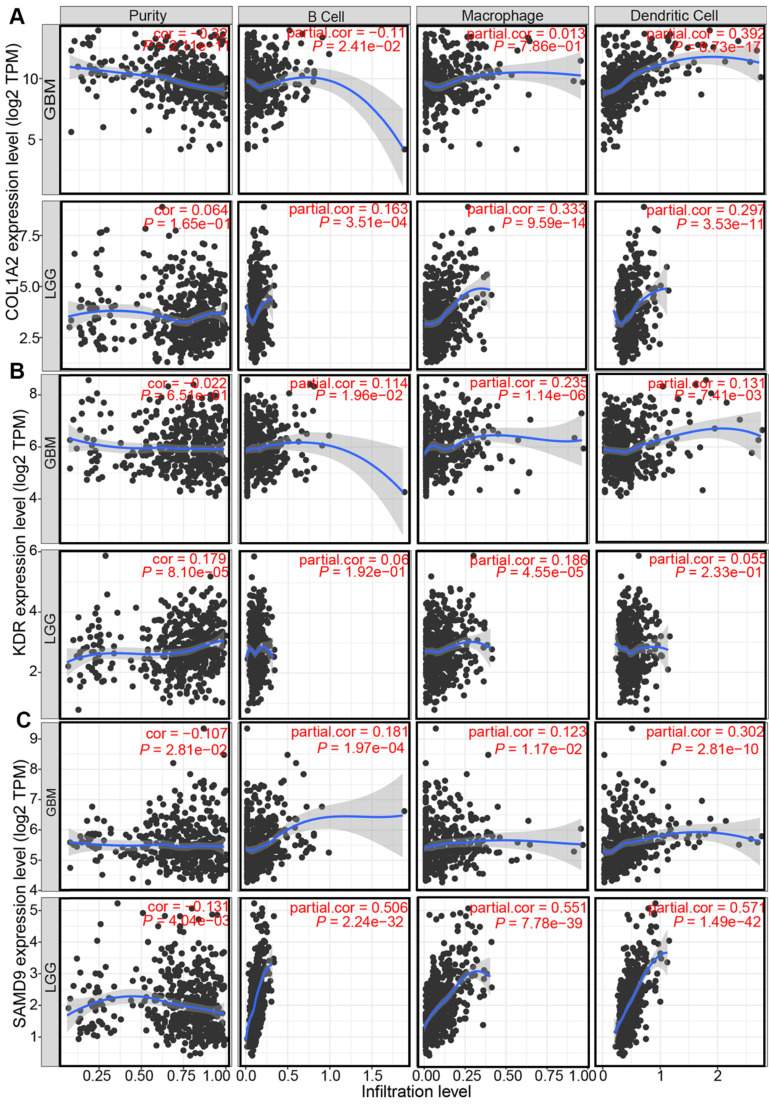
Correlation between the expression levels of COL1A2 (**A**), KDR (**B**), and SAMD9 (**C**) and infiltration of APCs in diffuse glioma.

**Figure 4:**
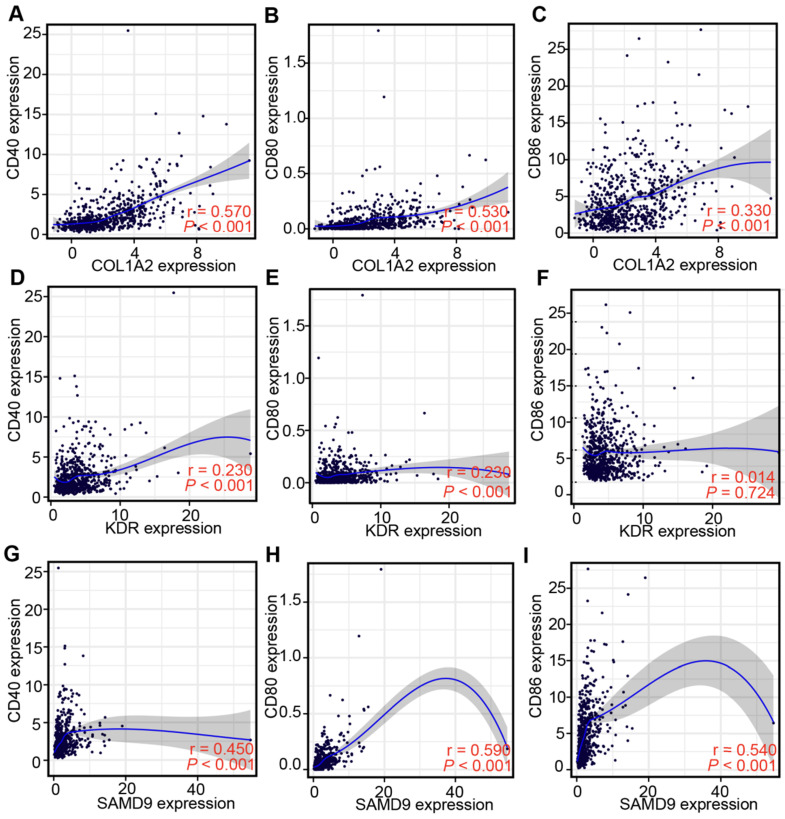
** Correlation between the expression levels of three antigens and those of APC markers in the TCGA diffuse glioma cohort. (A-C)** Correlation between the expression levels of COL1A2 and those of CD40 (A), CD80 (B), and CD86 (C). **(D-F)** Correlation between the expression levels of KDR and those of CD40 (D), CD80 (E), and CD86 (F). **(G-I)** Correlation between the expression levels of SAMD9 and those of CD40 (G), CD80 (H), and CD86 (I). Correlations were determined by the Spearman's test, and *P* < 0.05 was considered significant.

Professional APCs such as dendritic cells (DCs), B cells, and macrophages, can capture, process, and present allergens to cognate T cells 30, 31. The abundance of APCs was first obtained from TIMER to explore the potential relationship with the identified tumor antigens 24. In GBM, the mRNA expression of KDR and SAMD9 was found positively related to the abundance of B cells (KDR: r = 0.114; SAMD9: r = 0.181), macrophages (KDR: r = 0.235; SAMD9: r = 0.123), and DCs (KDR: r = 0.131; SAMD9: r = 0.302) (*P* < 0.05, Figure 3). However, only the mRNA expression of COL1A2 was significantly positively related to DC abundance(r = 0.392, *P* < 0.05, Figure 3). In the low-grade gliomas (LGGs), the mRNA expression of COL1A2 and SAMD9 was positively associated with the abundance of B cells (COL1A2: r = 0.163; SAMD9: r = 0.506), macrophages (COL1A2: r = 0.333; SAMD9: r = 0.551), and DCs (COL1A2: r = 0.297; SAMD9: r = 0.571) (*P* < 0.05, Figure 3). KDR expression was found significantly positively related to the abundance of macrophages(r = 0.182, *P* < 0.05) (Figure 3). The results suggested that mRNA vaccines targeting the three antigens might activate professional APCs to cognate T cells. Considering the synergistic role of certain helper stimulus molecules in the immune response, the association between the mRNA expression of KDR, COL1A2, and SAMD9 and that of CD80, CD86, and CD40 was analyzed 32, 33. We found the expression of COL1A2 and SAMD9 had moderately positive correlations with the expression of CD40 (COL1A2, r = 0.57; SAMD9, r = 0.45), CD80 (COL1A2, r = 0.53; SAMD9, r = 0.59), or CD86 (COL1A2, r = 0.33; SAMD9, r = 0.54) in the TCGA cohort (*P* < 0.05, Figure 4), which was validated in the CGGA cohort (Figure S1). KDR expression was positively associated with CD40 expression (r = 0.23, *P* < 0.05) and CD80 expression (r = 0.23, *P* < 0.05), but was not significantly related to CD86 expression(r = 0.014, *P* > 0.05) in the TCGA cohort (Figure 4). Our results also verified the relationship between KDR expression and CD40 expression(r = 0.39, *P* < 0.05) and CD80 expression (r = 0.40, *P* < 0.05) in the CGGA cohort (Figure S1). The relationship between KDR expression and CD86 (r = 0.28, *P* < 0.05) was observed in the CGGA cohort (Figure S1). Our results indicated that expression of the three antigens correlated with APC abundance and the expression of APC markers.

### Definitions of the three immune subtypes associated with the prognosis of diffuse glioma

**Figure 5:**
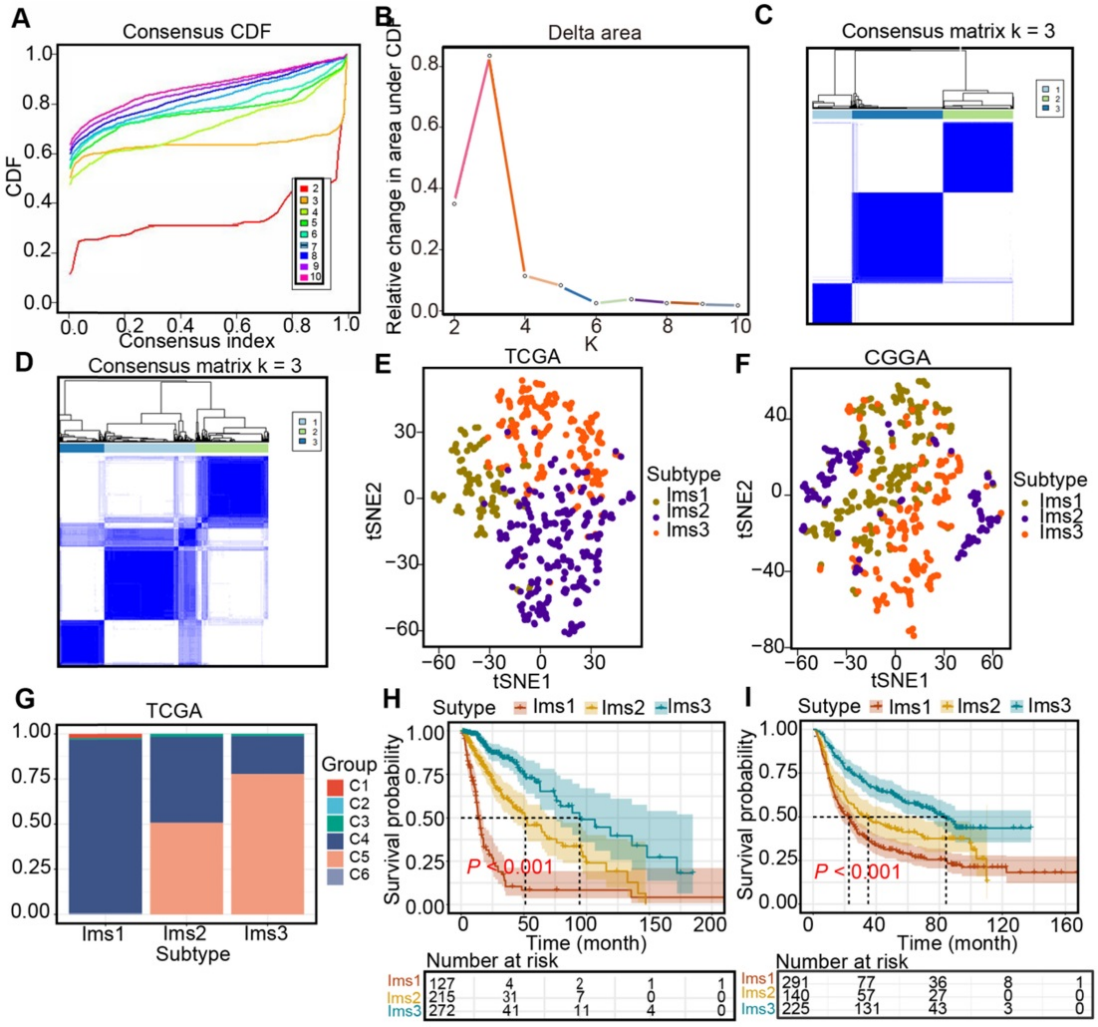
** Identification of immune subtypes in diffuse glioma. (A)** Cumulative distribution function (CDF) curve and **(B)** delta area in the TCGA cohort. Consensus scores for different subtype numbers (k = 2-10) are presented. **(C-D)** Heatmap representing the consensus matrix in the TCGA (C) and CGGA (D) cohorts. **(E-F)** Stratification into three subtypes validated by t-SNE analysis in the TCGA (E) and CGGA (F) cohorts. **(G)** Correlation between immune subtypes and six well-recognized pancancer immune subtypes in the TCGA cohort. **(H-I)** Survival analysis for three subtypes in the TCGA (H) and CGGA (I) cohorts. The log-rank test was used to determine the statistical significance of the differences, and *P* < 0.05 was considered significant.

Three immune-associated subtypes were defined by Consensus Cluster Plus analysis based on the immune gene profiles of the 3126 immune-related genes in the TCGA cohort 22. In the consensus clustering analysis, the consensus matrix (CM), delta area, and CFD curves were used to identify the optimal cluster number (n = 3) in the TCGA cohort (Figure 5A-C). t-SNE analysis was conducted to evaluate the subtype assignments, which indicated that the three subtypes of the samples were separated from each other (Figure 5D). Therefore, t-SNE analysis supported the classification into three subtypes. Similar results were observed in the CGGA cohort (Figure 5E-F). Finally, diffuse gliomas were classified into three subtypes (Ims1-3). In the TCGA cohort, the relationship between the classification in this study and those previously reported was explored. The results showed that diffuse gliomas with subtype Ims1 were mainly enriched in those with C4, those with subtype Ims2 were mainly enriched in C4, 5, and those with subtype Ims3 were mainly enriched in C5 (Figure 5G). Finally, the correlations between survival and the subtypes of diffuse gliomas were investigated. The findings demonstrated that patients with subtype Ims1 (n = 127, median survival time = 14.0 months) had shorter median survival times than those with Ims2 (n = 215, median survival time = 51.2 months) and Ims3 (n = 272, median survival time = 94.5 months) in the TCGA cohort (log-rank test, *P* < 0.05, Figure 5H). In addition, the prognosis of patients with Ims3 was better than those with Ims2 (log-rank test, *P* < 0.05). A similar prognostic difference was observed in the CGGA cohort (Ims1, n = 291, median survival time = 22.7 months; Ims2, n = 140, median survival time = 35.0 months; Ims3, n = 225, median survival time = 84.2 months, log-rank test, *P* < 0.05, Figure 5I). In conclusion, the three immune subtypes associated with the clinical outcomes of diffuse gliomas were defined.

### The clinical, cellular, and molecular characteristics of diffuse glioma with the three subtypes

Subsequently, the clinical features of the three subtypes were investigated, including age, sex, tumor grade, IDH mutation status, 1p19q codeletion status, and MGMT promoter status in the TCGA and CGGA cohorts. Diffuse glioma with the three subtypes showed distinct clinical features in both cohorts (Table 1). Malignant phenotypes such as higher histological grade and more wild-type IDH were enriched in patients with subtype Ims1 compared with the other subtypes (X^2^ test, FDR < 0.001). In addition, the 1p19q codeletion status and MGMT promoter status were significantly correlated with the three subtypes (X^2^ test, FDR < 0.001). The noncodeleted 1p19q and unmethylated MGMT promoters were enriched in patients with subtype Ims1 compared with those with subtypes Ims2 and Ims3 (X^2^ test, FDR < 0.001).

**Figure 6:**
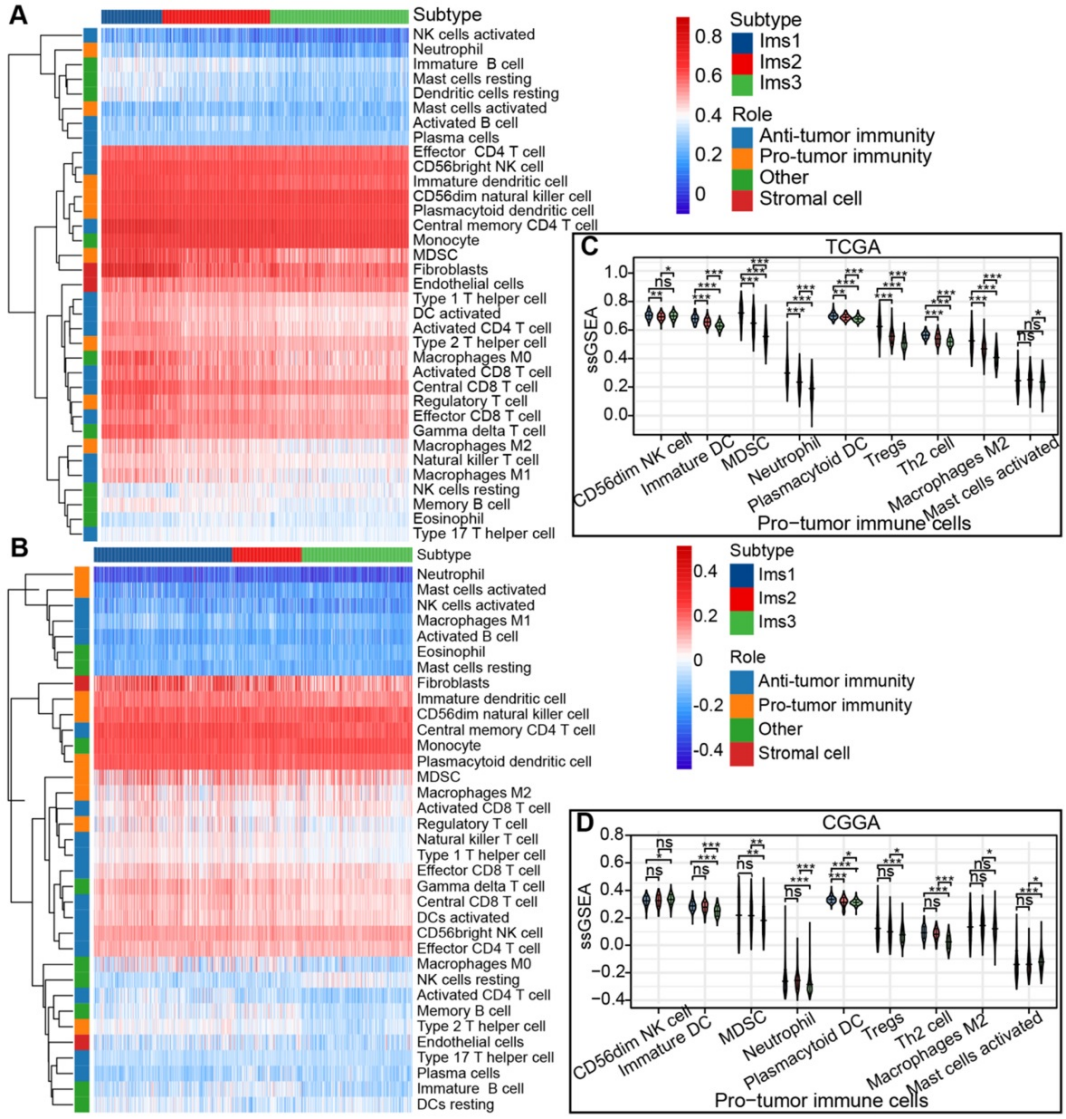
** The immune characteristics of diffuse glioma with three subtypes. (A-B)** Heatmap describing the abundance of protumor and antitumor immune cells, stromal cells, other cells of the three subtypes in the TCGA (A) and CGGA (B) cohorts. **(C-D)** Boxplot of the abundance of protumor immune cells distinguished by different subtypes in the TCGA (C) and CGGA (D) cohorts. The significant difference was compared by the Kruskal-Wallis test, and the *P* values are labeled above each boxplot with asterisks (ns represents no significance, * *P* < 0.05, ** *P* < 0.01, *** *P* < 0.001.

The ssGSEA algorithm was adopted to calculate the abundance of 35 cell types classified into protumor and antitumor immune cells, stromal cells, and others in the TCGA and CGGA cohorts to identify the subtypes suitable for immunotherapy targeting the three antigens and to characterize their immunologic landscape. The results are presented in a heatmap (Figure 6A-B). Most of the protumor immune cells (CD56dim NK cells, immature DCs, myeloid-derived suppressor cells (MDSCs), neutrophils, plasmacytoid DCs, Tregs, Th2 cells, and M2 macrophages) were enriched in subtype Ims1 compared to the other subtypes (*P* < 0.05, Figure 6C), suggesting that subtype Ims1 was characterized by the protumor microenvironment in diffuse gliomas. A similar significant difference was observed in the CGGA cohort (*P* < 0.05, Figure 6D). The results were consistent with previously reported immune subtypes (C1-C6), among which C4 was enriched in gliomas' more prominent macrophage signature 34. Similar to protumor immune cells, most immune checkpoints (such as PD-1, CD40, PD-L1, CD80, and CD86) were highly expressed in subtype Ims1 in the TCGA cohort compared with the other subtypes (*P* < 0.05, Figure S2A), which was consistent with the results in the CGGA cohort (*P* < 0.05, Figure S2B). In addition, more than half of the immunogenic cell death (ICD) modulators (11/21), such as FPR1, CXCL10, ANXA1, and MET, were highly expressed in subtype Ims1 compared with the other subtypes in the TCGA and CGGA cohorts (*P* < 0.05, Figure S2C-D). Our identified immune subtypes could distinguish the expression levels of immune checkpoints and ICD modulators.

**Figure 7:**
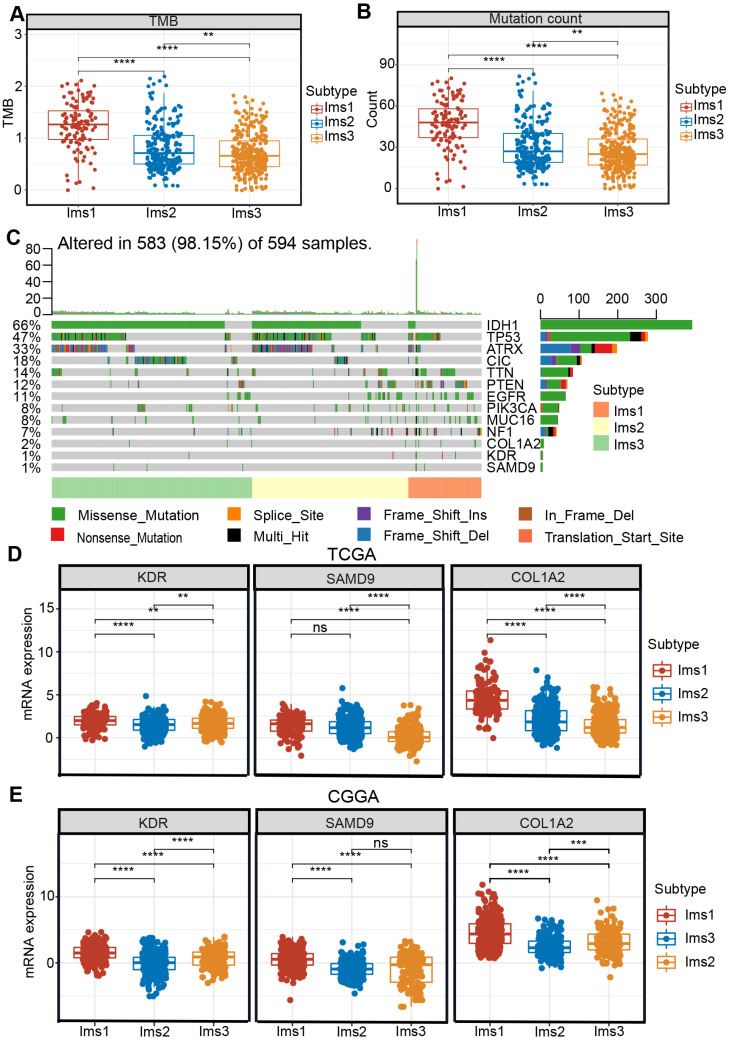
** Correlation between immune subtypes and tumor mutational burden (TMB), mutation and three antigens. (A-B)** TMB (A) and number of mutated genes (B) evaluated in the three subtypes. **(C)** The landscape of 10 genes with the most frequent genomic alteration and three antigens. **(D-E)** The mRNA expression of three antigens evaluated in the three subtypes in the TCGA (D) and CGGA (E) cohorts. The significant difference was compared by the Kruskal-Wallis test, and the P values are labeled above each boxplot with asterisks (ns represents no significance, ** *P* < 0.01, *** *P* < 0.001).

Studies have shown that acquired mutations might create neoantigens that affect the patients' response to immunotherapy 35. After excluding samples with extreme deviate values using the “outliers” package (Figure S3-4), diffuse glioma with subtype Ims1 was found to have a significantly higher TMB and mutation count than subtype Ims2 and Ims3 (*P* < 0.05, Figure 7A-B). The landscape of the ten genes with the most frequent genomic alterations and the three antigens was displayed across gliomas in the TCGA cohort (Figure 7C). In particular, diffuse gliomas with subtype Ims1 had more infrequent IDH mutations and more frequent PTEN and NF1 mutations (Figure 7C). The three genes were mutated frequently in subtype Ims1, suggesting gliomas with subtype Ims1 were more likely to produce tumor neoantigens. Therefore, the immune subtypes reflected the immune status and could help identify suitable patients for mRNA vaccination. The expression of the three antigens in the three subtypes was evaluated, revealing that subtype Ims1 consistently exhibited higher expression of KDR, COL1A2, and SAMD9 than subtype Ims2 and Ims3 (*P* < 0.001, Figure 7D-E). Overall, diffuse gliomas with immune subtypes exhibited distinct clinical, cellular, and molecular characteristics. Patients with subtype Ims1 might be more suitable for mRNA vaccination targeting KDR, COL1A2, and SAMD9.

### The association between the expression of three antigens and APC markers in diffuse glioma with subtype Ims1

**Figure 8:**
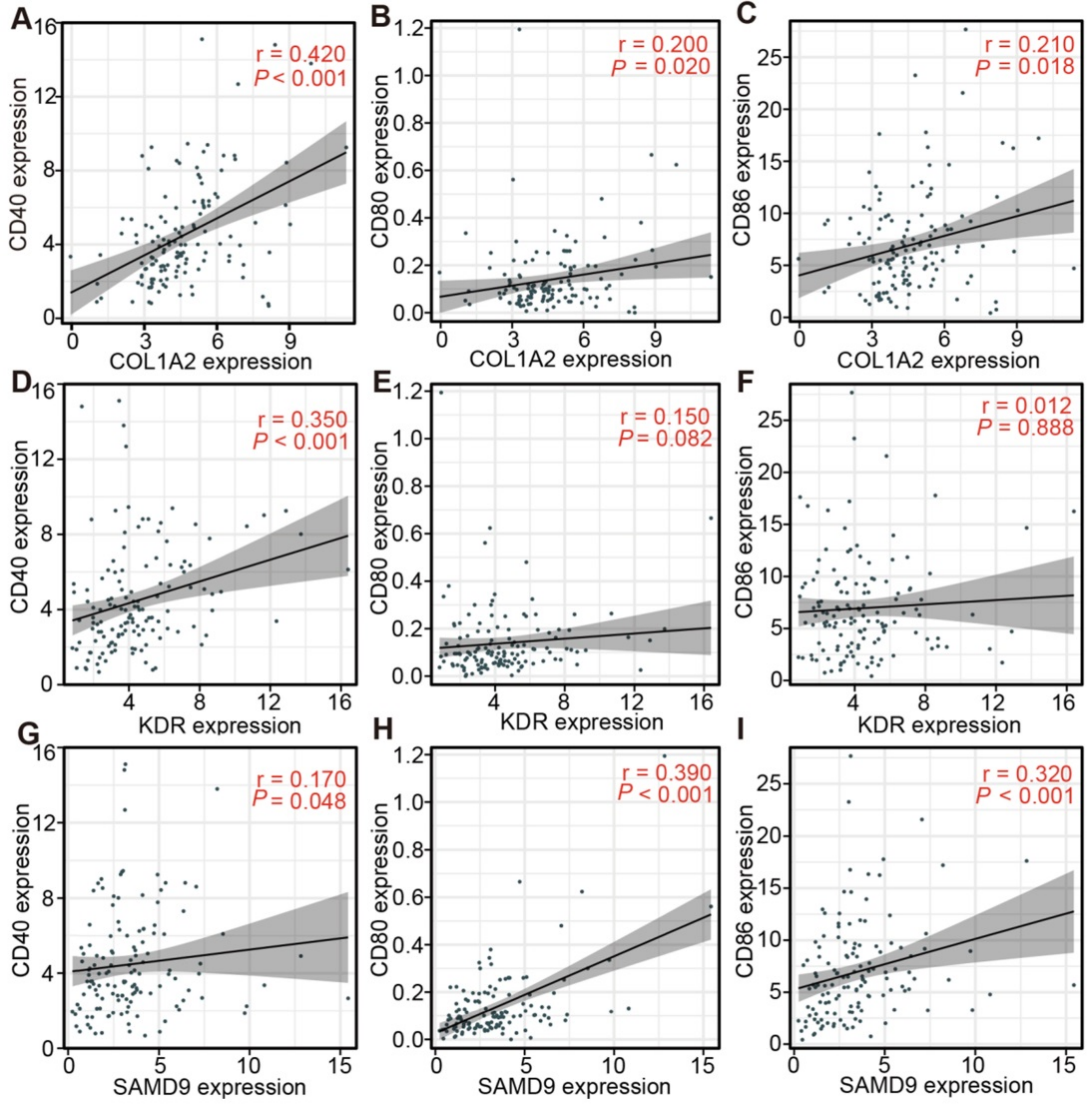
** Correlation between the expression levels of three antigens and those of APC markers in diffuse glioma with subtype Ims1 in the TCGA cohort. (A-C)** Correlation between the expression levels of COL1A2 and those of CD40 (A), CD80 (B), and CD86 (C). **(D-F)** Correlation between the expression levels of SAMD9 and those of CD40 (D), CD80 (E), and CD86 (F). **(G-I)** Correlation between the expression levels of KDR and those of CD40 (G), CD80 (H), and CD86 (I). Correlations were determined by the Spearman rank test, and *P* < 0.05 was considered significant.

The relationship between the expression of the three antigens and APC markers in the TCGA and CGGA cohorts was analyzed to assess the efficacy of the immunogenicity targeting the three antigens for the mRNA vaccine of diffuse glioma with subtype Ims1. The results indicated that COL1A2 expression was positively associated with the mRNA expression of CD40, CD80, and CD86 in the TCGA (CD40, r = 0.42; CD80, r = 0.20; and CD86, r = 0.21) (Figure 8A-C) and CGGA cohorts (CD40, r = 0.64; CD80, r = 0.55; and CD86, r = 0.52) (Figure S5A-C). In addition, the relationship between SAMD9 expression and the mRNA expression of CD40 (TCGA, r = 0.17; CGGA, r= 0.41), CD80 (TCGA, r = 0.39; CGGA, r = 0.55), and CD86 (TCGA, r = 0.32; CGGA, r = 0.53) was similar to that of COL1A2 in the TCGA (Figure 8D-F) and CGGA cohorts (Figure S5D-F). These results suggested that the mRNA vaccine targeting COL1A2 and SAMD9 would enhance the antigen presentation of APCs. KDR expression had a positive association with CD40 expression in the TCGA (r = 0.35) and CGGA (r = 0.15) cohorts (Figure 8G, Figure S5G, *P* < 0.05). In addition, KDR expression was associated positively with CD80 expression in the CGGA cohort (r = 0.21) (Figure S5H, *P* < 0.05). But we did not find significant association between KDR expression and CD80 expression in the TCGA cohort (Figure 8H, *P* > 0.05). Unsatisfactorily, no statistically significant association between KDR expression and CD86 expression in the TCGA and CGGA cohorts was observed (Figure 8I, Figure S5I, *P* > 0.05). In conclusion, the expression of three antigens, especially COL1A2 and SAMD9, was closely associated with APC markers of diffuse glioma with subtype Ims1.

### The potential biological mechanisms of the three tumor antigens in diffuse gliomas

**Figure 9:**
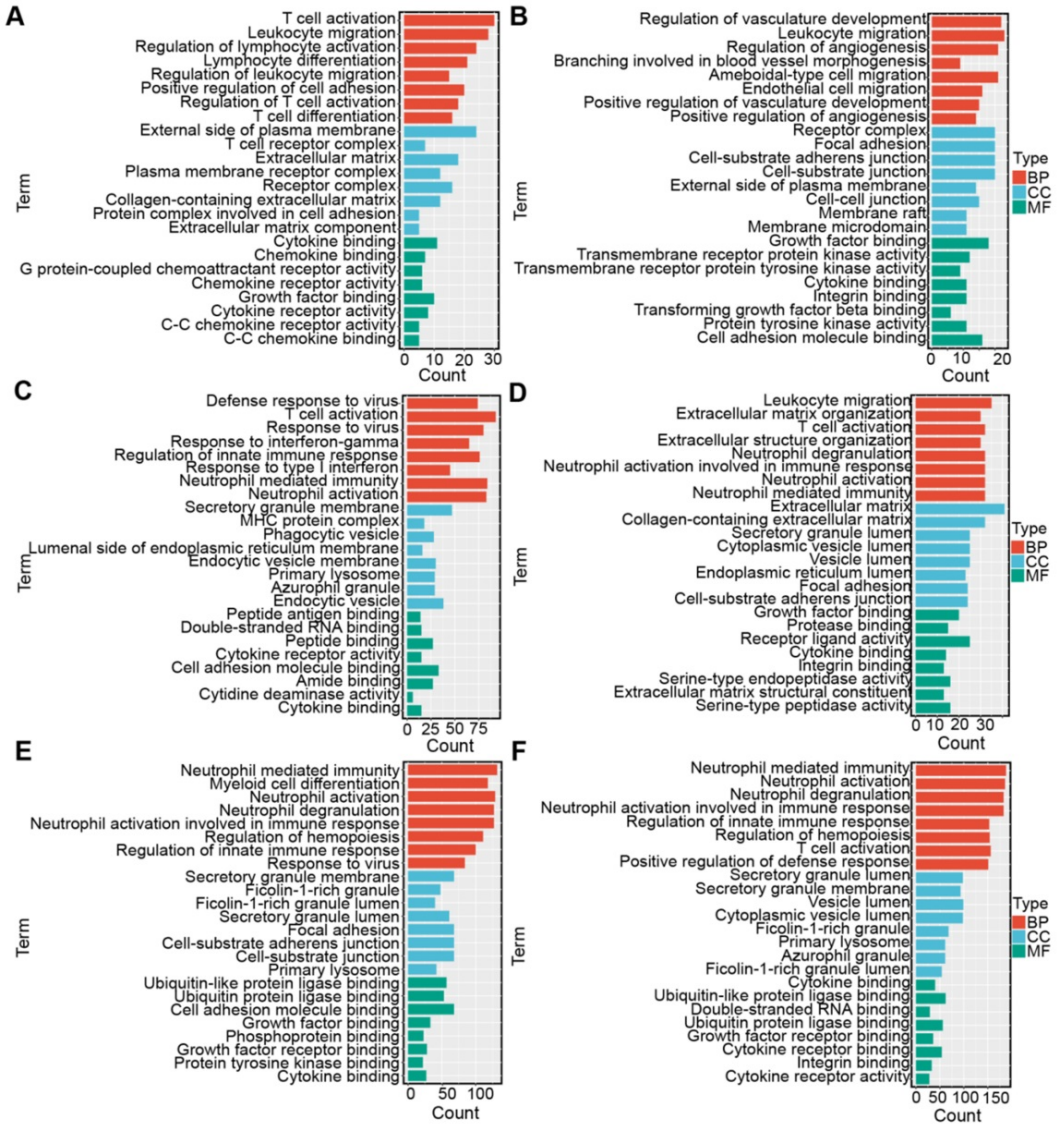
** Functional enrichment analysis. (A-C)** Gene ontology (GO) analysis for categories associated with COL1A2 (A), KDR (B), and SAMD9 (C) in the TCGA cohort. **(D-F)** GO analysis for categories associated with COL1A2 (D), KDR (E), and SAMD9 (F) in the CGGA cohort.

Correlation analysis was conducted to select the genes positively correlated with the three antigens (r > 0.3, *P* < 0.001) from the 3192 immune genes in the TCGA and CGGA cohorts so that the underlying immune-associated pathways of the three antigens could be identified. The most highly enriched pathways of COL1A2 included immune-related pathways, such as “T cell activation”, “leukocyte migration” and “lymphocyte migration” in the TCGA and CGGA cohorts (Figure 9A, D). According to the GO biological process (BP) enrichment analysis of KDR, we found that most of the highly enriched biological pathways included “regulation of innate immune response” and “leukocyte migration”, as respectively shown in the TCGA (Figure 9B) and CGGA cohorts (Figure 9E). Similar results of functional enrichment analysis of SAMD9 were observed in the TCGA and CGGA cohorts. SAMD9-positive-related genes were enriched in pathways such as “T cell activation” and “regulation of innate immune response” in the TCGA and CGGA cohorts (Figure 9C, F). These results indicated that COL1A2, KDR, and SAMD9 were involved in immunologic biological processes of diffuse glioma, providing a theoretical possibility for mRNA vaccine development.

### Identification of the subtype- and immune-associated hub modules of diffuse glioma

**Figure 10:**
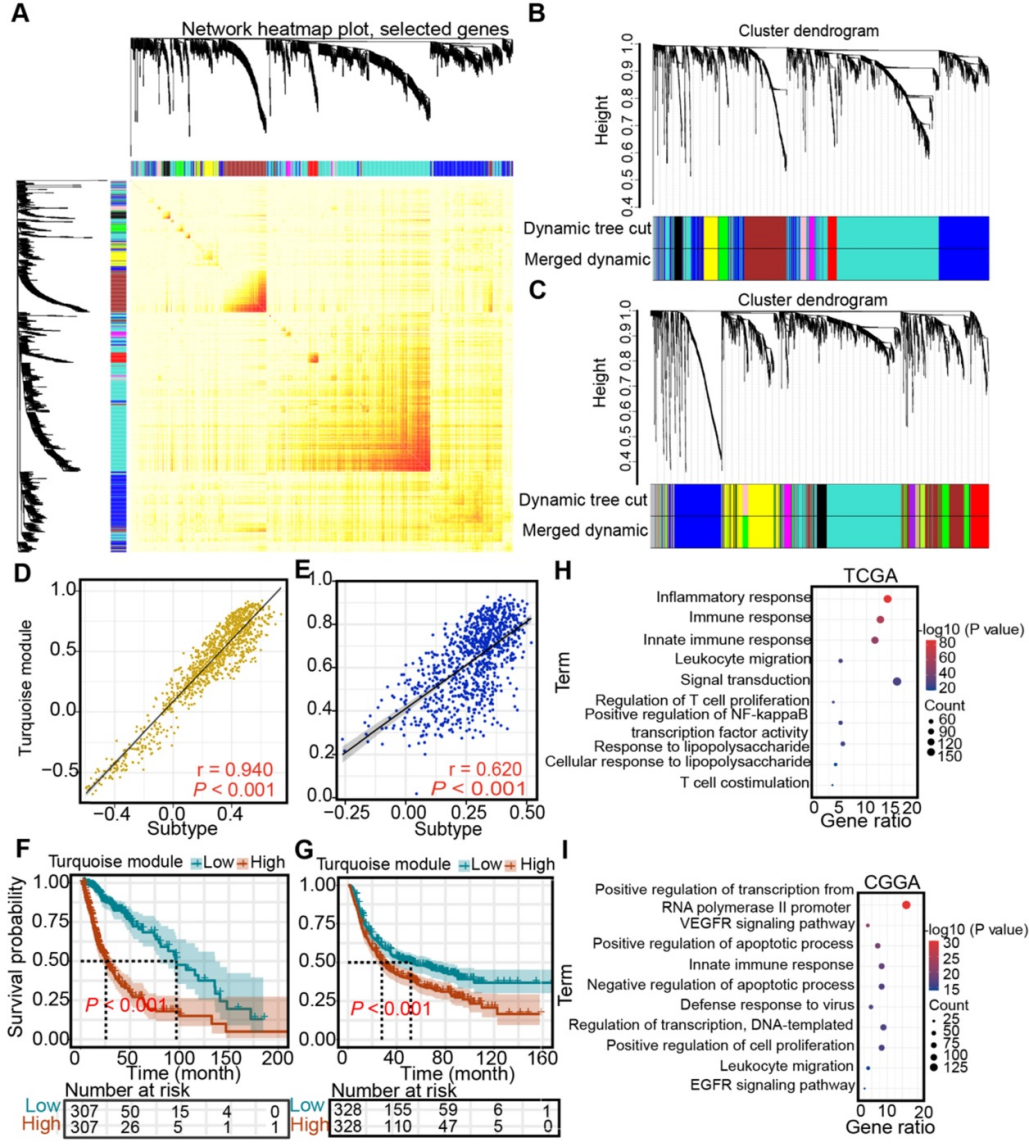
** Identification of subtype-associated modules and antigens. (A)** Topological overlap matrix plot. **(B-C)** Dendrogram of immune genes clustered based on a dissimilarity measure in the TCGA (B) and CGGA (C) cohorts. **(D-E)** Correlation between the turquoise module and immune subtype in the TCGA (D) and CGGA (E) cohorts. Correlations were determined by the Pearson test, and *P*< 0.05 was considered significant. **(F-G)** Survival analysis of the turquoise module in the TCGA (F) and CGGA (G) cohorts. The log-rank test was used to determine the statistical significance of the differences, and *P* < 0.05 was considered significant. **(H-I)** GO analysis for hub genes from the turquoise module in the TCGA (H) and CGGA cohorts (I).

Next, WGCNA was performed to identify the subtype- and immune-associated hub modules and genes. In the TCGA and CGGA cohorts, we uncovered that the turquoise module was closely associated with immune subtypes (Figure 10A-C), which was validated by correlation analysis (TCGA: r = 0.94; CGGA: r = 0.62) (Figure 10D-E). Then, the prognostic significance of hub gene signature expression was calculated by the average expression of 1105 genes from a turquoise module in the TCGA cohort and 865 hub genes from a turquoise module in the CGGA cohort. Higher hub gene expression was associated with worse overall survival (log-rank test, *P* < 0.05, Figure 10F-G). Finally, the pathways highly enriched with these hub related genes were explored. In the TCGA cohort, the most highly enriched pathways of the hub genes clustered into turquoise modules included immune-related pathways, such as “inflammatory response”, “immune response”, “innate immune response”, and “leukocyte migration” (Figure 10H). Similarly, the most highly enriched pathways in the CGGA cohort were related to immunologic processes, such as “innate immune response”, and “leukocyte migration” (Figure 10I).

## Discussion

Diffuse glioma is one of the most aggressive malignancies, and is characterized by biological and prognostic heterogeneity. Despite aggressive treatment, the prognosis remains unsatisfactory. Combined with the considerable success of immunotherapy for cancer, the profiles of over-expressed, mutated, and amplified genes were constructed to improve patient prognosis and provide more treatment options for patients (Figure 1). Proteins encoded by genes that are qualitatively or quantitatively altered differ from normal proteins, which are recognized by immune cells to kill cancer cells and could serve as tumor antigens 11. In our identified potential antigens, we found that mRNA expression of KDR, COL1A2, and SAMD9 was significantly associated with worse prognosis in patients with diffuse glioma in the TCGA and CGGA cohorts (Figure 2), indicating that the development of mRNA vaccines targeting these three antigens may prolong the survival time of patients.

Antigens are recognized by naive T cells, leading to attacks against cancer cells depending on the capture, processing, and presentation of APCs. DCs, B cells, and macrophages are specialized APCs that initiate and regulate innate and adaptive immune responses 30, 31. Some studies have recently shown that numerous immune cells, such as macrophages and dendritic cells, can be found in the central nervous system. The correlation of antigen expression and APC infiltration helps identify which APCs are activated by the antigen, providing support for potential antigen identification. Our findings demonstrated that COL1A2 and SAMD9 expression was positively associated with high APC infiltration, especially DCs, in GBM (COL1A2, r = 0.392; SAMD9, r = 0.302) and LGG (COL1A2, r = 0.297; SAMD9, r = 0.571) (Figure 3). KDR expression was positively correlated with macrophage abundance (LGG, r = 0.182; GBM, r = 0.235) (Figure 3). The presentation of these antigens to T cells depends on different APCs to support the individualized selection of mRNA vaccines. In addition, the mRNA expression of KDR, COL1A2, and SAMD9 had a positive, although not completely consistent, relationship with the mRNA expression of APC markers, such as CD40, CD80, and CD86, in the TCGA and CGGA cohorts (Figure 4, Figure S1), validating the close relationship between antigens and APCs. CD40, CD80, and CD86 are constitutively expressed in APCs and are critical for the response to T-dependent antigens. This positive relationship enhances the activation of T cells and induces severe immune attacks 32, 33.

These results suggested that COL1A2, KDR, and SAMD9 play vital roles in immunity as potential antigens. The genes related to COL1A2, KDR, and SAMD9 were highly enriched in immune-related pathways, such as “T cell activation”, “leukocyte migration”, “regulation of innate immune response” and “lymphocyte migration” (Figure 10). Our results were consistent with recent studies. KDR was highly expressed highly in GBM and associated with unfavorable prognosis for GBM patients, as validated in the TCGA and CGGA cohorts (Figure 1-2). The deletion of KDR in DCs can reduce type I interferon, which is the immunomodulatory effect of KDR expression 36. In fact, the mRNA expression of KDR was positively associated with the abundance of DCs (r = 0.131, *P* < 0.05, Figure 3), further revealing that KDR serves as an antigen that activates APCs, especially DCs.

Numerous studies have shown that COL1A2 is closely related to the prognosis of patients with gastric cancer 37, and lung and esophageal cancers 38. In gastric cancer, COL1A2 is positively correlated with lymphatic metastasis, and macrophages, which is consistent with our results in low-grade glioma (r = 0.333, *P* < 0.05, Figure 3). SAMD9 is significantly up-regulated in glioma, and its expression is positively correlated with tumor grade 39. In the measles virus-mediated treatment of GBM, SAMD9 serves as an innate antiviral host factor in response to IFN stimulation and is involved in death signaling 40. These findings altogether support our results. However, the regulation of COL1A2, SAMD9, and KDR in immunity in diffuse glioma remains unclear.

Because therapeutic effects vary for patients, three immune-associated subtypes were identified according to the immune gene profile to identify which subtype was suitable for immunotherapy targeting three antigens. The three immune-associated subtypes exhibited distinct prognostic molecular, cellular, and clinical characteristics in the TCGA and CGGA cohorts. Diffuse gliomas with subtype Ims1 had a poorer prognosis than gliomas with the other subtypes (Figure 5H-I). In addition, malignant phenotypes such as higher histological grade and more wild-type IDH were enriched in patients with subtype Ims1 compared with the other subtypes (X^2^ test, FDR < 0.05)(Table 1). Diffuse gliomas with subtype Ims1 were mainly enriched in those with previously reported C4 (lymphocyte depleted) 34 and displayed the highly abundant protumor cells, such as M2 macrophages (Figure 6C-D). Similarly, C4 is also rich in particular glioma subtypes and displays a high M2 response 34. Although diffuse glioma with subtype lms1 has higher levels of TMB, tumor antigens, and APC activation markers, it has worse survival. As shown in the literature 41, 42, diffuse glioma with high-level TMB has a significantly different prognosis of 14 out of 20 cancer types. Diffuse gliomas with subtype Ims1 displayed higher levels of TMB and mutation number and had more infrequent IDH mutations and more frequent PTEN and NF1 mutations. Glioma with wild-type IDH and mutant PTEN and NF1 has poor prognosis 43, 44. High tumor mutation burden has been proposed as a predictive biomarker for response to immune checkpoint blockade (ICB), largely due to the potential for tumor mutations to generate immunogenic neoantigens 45, 46. Glioma with a high level of TMB may produce high level of tumor antigens that can activate APC infiltration. Therefore, the Ims1 subtype with high levels of TMB displays high levels of tumor antigens and APCs. Tumor antigens are recognized and processed by APCs. Then, the processed tumor antigens are presented to helper T cells, which secrete cytokines to promote the proliferation of killer T cells and produce specific killing effects. Infiltration of T cells, especially CD8+ T cells, into the tumor microenvironment correlates with better prognosis in brain cancer 47. However, even when tumor-specific CD8+ T cell responses are observed, they rarely provide protective immunity as tumors often evade immune surveillance by dampening T cell effector and memory functions 48, 49. The highly abundant protumor cells and high expression of immune checkpoints in subtype Ims1 suggested an immunosuppressive glioma microenvironment in the TCGA and CGGA cohorts (Figure S2A-B), which could inhibit the effective immune response. However, in the clinical trials, mRNA vaccines combined with checkpoint inhibitors may improve the effective immune response 50.

Subtype Ims1 had a higher TMB and mutation counts and could drive more neoantigen-specific T cell infiltration (Figure 7A-B), which is closely associated with immunotherapeutic efficacy 26, 35, 42. KDR, COL1A2, and SAMD9 were frequently mutated and highly expressed in gliomas with subtype Ims1 (Figure 7C-E). Therefore, we showed diffuse glioma with subtype Ims1 is suitable for immunotherapy, which was supported by the close association between the expression of most APC markers and that of the three antigens, especially SAMD9 (Figure 8, Figure S5).

In addition, most tumor antigens exhibit immunogenicity that is too weak to elicit an effective antitumor immune response. The immune response to tumor antigens leads to the reduction or loss of tumor surface antigens, so that tumor cells are not recognized by the immune system and escape immune attack (antigen modulation) 51. Tumor antigens may be coated with substances, such as salivary mucopolysaccharides, that are not recognized by the host's lymphocytes and thus cannot induce tumor cell killing effect. Therefore, when endogenous tumor antigens fail to elicit an effective immune response, it is necessary to reactivate and modulate the immune response through exogenous vaccines.

In this study, COL1A2, SAMD9, and KDR were identified as promising antigens, and patients with corresponding immune subtypes are suitable for mRNA vaccine treatment. Nevertheless, these three antigens need to be validated in future studies.

## Conclusions

In conclusion, COL1A2, SAMD9, and KDR are the potential antigens for mRNA vaccine development, which could benefit patients with Ims1 diffuse glioma. The findings in this study provide a theoretical foundation for developing mRNA vaccines against diffuse glioma, predicting patient prognosis, and defining the suitable patients for vaccination.

## Supplementary Material

Supplementary figures and tables.

## Figures and Tables

**Table 1:** Association of three subtypes with clinical characteristics in the TCGA and CGGA cohorts

Characteristic	The TCGA cohort (n, %)				The CGGA cohort (n, %)			
	Ims1 (133,19.8)	Ims2 (236,35.1)	Ims3 (303,45.1)	P value	Ims1 (301,43.4)	Ims2 (151,21.8)	Ims3 (241,34.8)	P value
**Age**				<0.001				<0.01
≤40y	21 (15.8)	101 (42.8)	144 (47.5)		109 (36.2)	60 (39.7)	122 (50.6)	
>40y	112 (84.2)	134 (56.8)	158 (52.1)		192 (63.8)	91 (60.3)	119 (49.4)	
N/A	0 (0)	1 (0.4)	1 (0.3)		0 (0)	0 (0)	0 (0)	
**Gender**				0.369				0.396
Female	50 (37.6)	94 (39.8)	140 (46.2)		122 (40.5)	62 (41.1)	111 (46.1)	
Male	83 (62.4)	141 (59.7)	162 (53.5)		179 (59.5)	89 (58.9)	130 (53.9)	
N/A	0 (0)	1 (0.4)	1 (0.3)		0 (0)	0 (0)	0 (0)	
**Histology**				<0.001				<0.001
Astrocytoma	10 (7.5)	99 (42.0)	83 (27.4)		103 (34.2)	59 (39.1)	109 (45.2)	
Oligodendroglioma	7 (5.3)	46 (19.5)	137 (45.2)		43 (14.3)	29 (19.2)	70 (29.0)	
Oligoastrocytoma	3 (2.3)	49 (20.8)	60 (19.8)		0 (0)	11 (7.3)	19 (7.9)	
Glioblastoma	113 (85.0)	37 (15.7)	10 (3.3)		155 (51.5)	52 (34.4)	42 (17.4)	
N/A	0 (0)	2 (0.8)	12 (4.0)		0	0	1 (0.4)	
**Grade**				<0.001				<0.001
G2	1 (0.8)	81 (34.3)	166 (54.8)		50 (16.6)	52 (34.4)	86 (35.7)	
G3	19 (14.3)	117 (49.6)	126 (41.6)		96 (31.9)	47 (31.1)	112 (46.5)	
G4	113 (85)	37 (15.7)	10 (3.3)		155 (51.5)	52 (34.4)	42 (17.4)	
N/A	0 (0)	1 (0.4)	1 (0.3)		0 (0)	0 (0)	1 (0.4)	
**IDH status**				<0.001				<0.001
Mutant	12 (9.0)	155 (65.7)	258 (85.1)		113 (37.5)	89 (58.9)	154 (63.9)	
WT	115 (86.5)	79 (33.5)	44 (14.5)		187 (62.1)	61 (40.4)	38 (15.8)	
N/A	6 (4.5)	2 (0.8)	1 (0.3)		1 (0.3)	1 (0.7)	49 (20.3)	
**1p/19q codeletion**				<0.001				<0.001
Non-codel	129 (97.0)	200 (84.7)	169 (55.8)		258 (85.7)	67 (44.4)	153 (63.5)	
Codel	1 (0.8)	3 (1.3)	134 (44.2)		37 (12.3)	24 (15.9)	84 (34.9)	
N/A	3 (2.3)	3 (1.3)	0 (0)		6 (2)	60 (39.7)	4 (1.7)	
**MGMT promoter status**				<0.001				<0.001
Unmethylated	59 (44.4)	68 (28.8)	37 (12.2)		112 (37.2)	38 (25.2)	77 (32.0)	
Methylated	48 (36.1)	162 (68.6)	264 (87.1)		118 (39.2)	68 (45.0)	129 (53.5)	
N/A	26 (19.5)	6 (2.5)	2 (0.6)		1 (0.3)	45 (30.0)	35 (14.5)	

## Abbreviations

1p19q: chromosome arms 1p and 19q
APC: antigen-presenting cell
BP: biological processes
CC: cellular components
CDF: Cumulative distribution function curves
CGGA: Chinese Glioma Genome Atlas
CM: consensus matrix
CNV: copy number variation
DAVID: Database for Annotation, Visualization, and Integrated Discovery
DCs: dendritic cells
DSS: disease-specific survival
GBM: glioblastoma
GEPIA: Gene Expression Profiling Interactive Analysis
GO: Gene Ontology
GTEx: Genotype-Tissue Expression
ICD: immunogenic cell death
IDH1: isocitrate dehydrogenase 1
IDH2: isocitrate dehydrogenase 2
LGGs: low-grade gliomas
MDSCs: myeloid-derived suppressor cells
MF: molecular function
MGMT: O(6)-methylguanine-DNA methyltransferase
OS: overall survival
PFI: progression-free interval
ssGSEA: single-sample Gene Set Enrichment Analysis
t-SNE: t-distributed stochastic neighbor embedding
TCGA: the Cancer Genome Atlas
TIMER: Tumor Immune Estimation Resource
TMB: tumor mutational burden
TMZ: temozolomide
WGCNA: Weighted gene coexpression network analysis
WHO: World Health Organization
