# Supplementary material for: Identification of three tumor antigens and immune subtypes for mRNA vaccine development in diffuse glioma
Source: Theranostics. 2021 Oct 3;11(20):9775–90. doi: 10.7150/thno.61677 (PMC8581422; doi:10.7150/thno.61677)
Supplement: Supplementary file 1 — Supplementary figures and tables. [file thnov11p9775s1.pdf]

Supplementary material

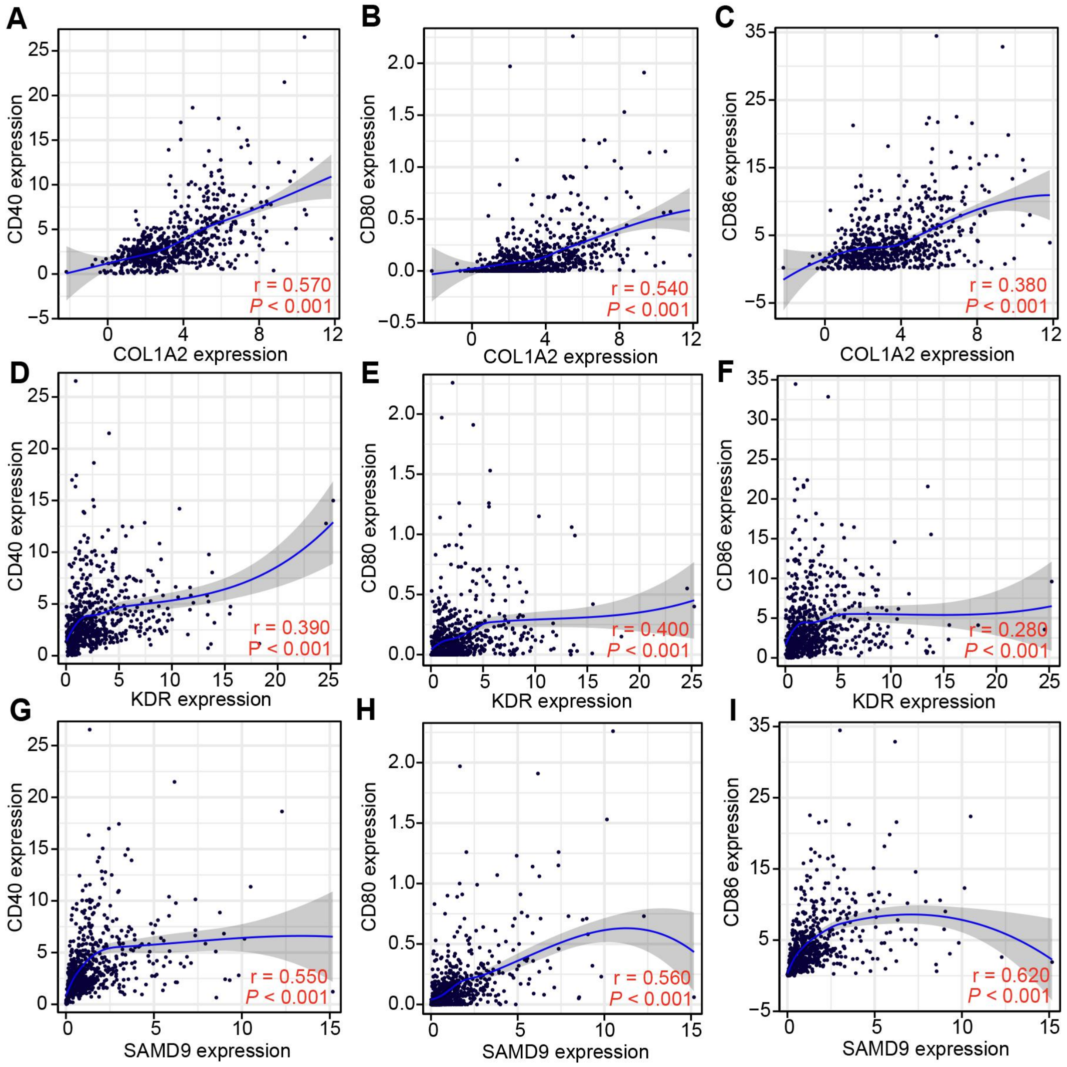

Figure S1. Correlation between the expression levels of three antigens and those of APC markers in the CGGA diffuse glioma. (A-C) Correlation between the expression levels of COL1A2 and those of CD40 (A), CD80 (B), and CD86 (C). (D-F) Correlation between the expression levels of KDR and those of CD40 (D), CD80 (E), and CD86 (F). (G-I) Correlation between the expression levels of SAMD9 and those of CD40 (G), CD80 (H), and CD86 (I). Correlations were determined by the Spearman' test, and $P < 0.05$

was considered significant.

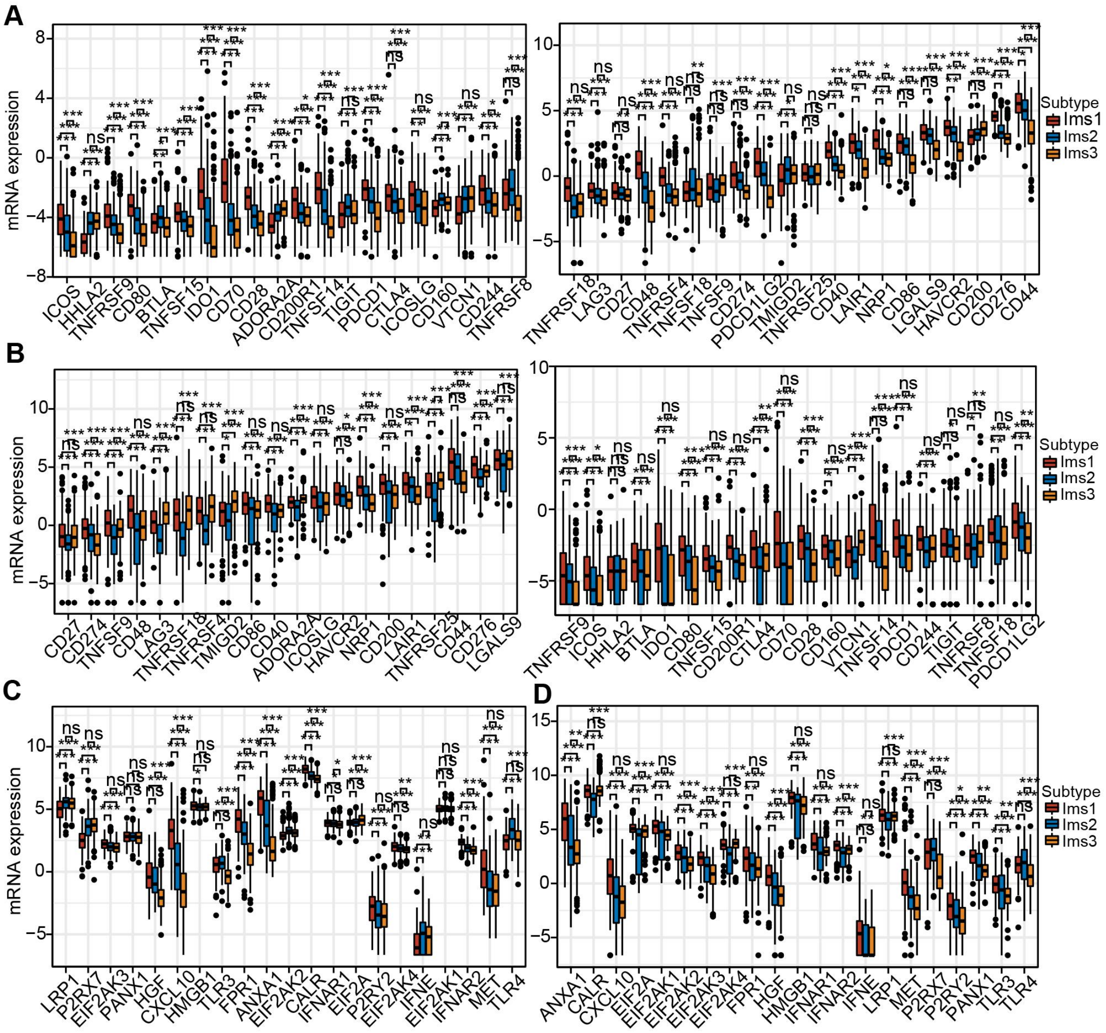

Figure S2. The expression levels of immune checkpoint genes and immunogenic cell death modulators in the three subtypes of diffuse gliomas. (A-B) Boxplot of mRNA expression of immune checkpoint genes of three subtypes in the TCGA (A) and CGGA (B) cohorts. (C-D) Boxplot of mRNA expression of immunogenic cell death modulators of three subtypes in the TCGA (C) and CGGA (D) cohorts. The significant difference was compared by the Kruskal-Wallis test, and the *P* values are labeled above each

boxplot with asterisks (ns represents no significance, * $P < 0.05$, ** $P < 0.01$, *** $P < 0.001$).

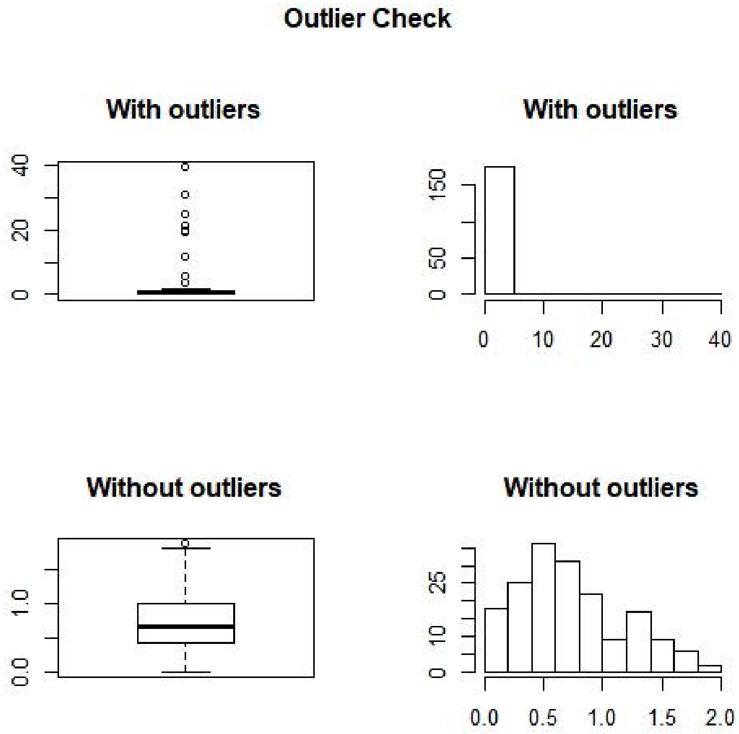

Figure S3. TMB distribution before and after excluding sample outliers.

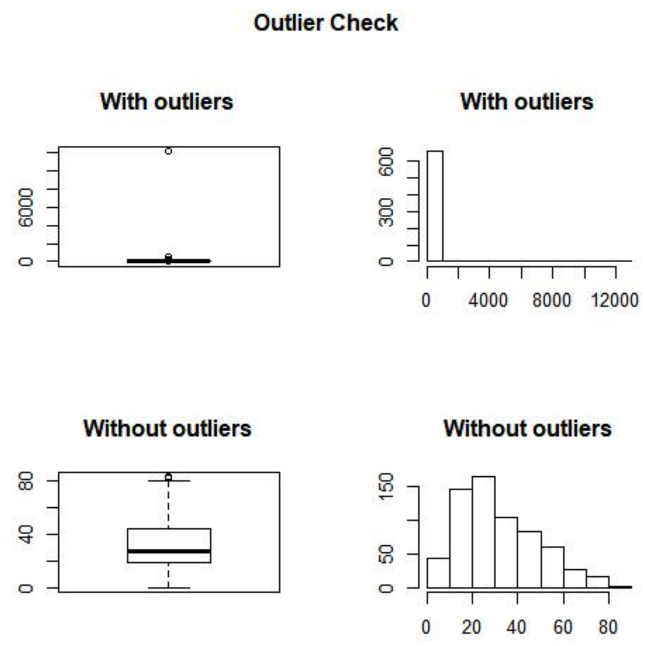

Figure S4. Mutation count distribution before and after excluding sample outliers.

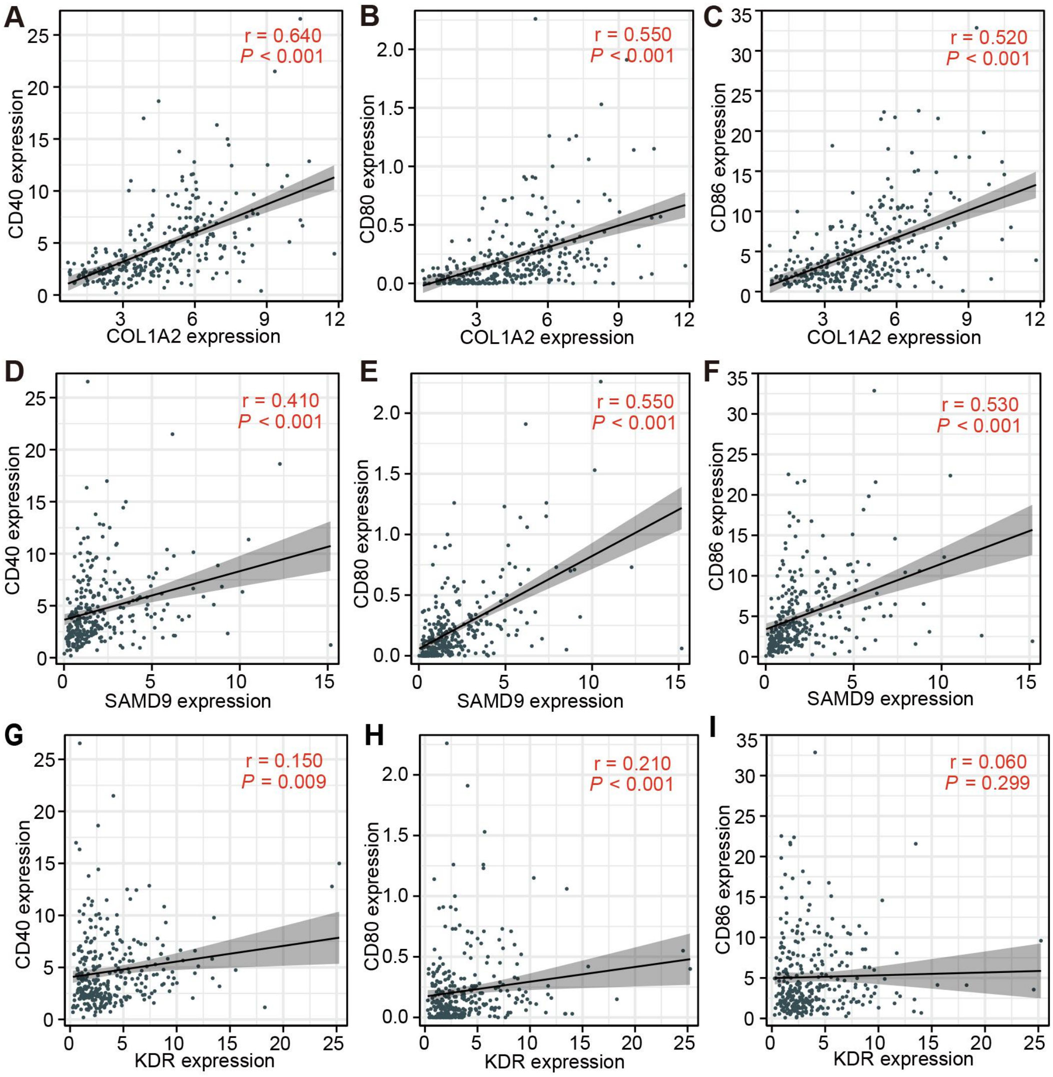

Figure S5. Correlation between the expression levels of three antigens and those of APC markers in diffuse glioma with subtype Ims1 in the CCGA cohort. (A-C) Correlation between the expression levels of COL1A2 and those of CD40 (A), CD80 (B), and CD86 (C). (D-F) Correlation between the expression levels of KDR and those of CD40 (D), CD80 (E), and CD86 (F). (G-I) Correlation between the expression levels of SAMD9 and those of CD40 (G), CD80 (H), and CD86 (I). Correlations were determined by the Spearman rank test, and $P < 0.05$ was considered

significant.
